# Heterogeneous Trajectory Classes of Social Engagement and Sex Differences for Older Adults in China

**DOI:** 10.3390/ijerph17228322

**Published:** 2020-11-11

**Authors:** Linglong Ye, Jian Xiao, Ya Fang

**Affiliations:** 1 State Key Laboratory of Molecular Vaccinology and Molecular Diagnostics, School of Public Health, Xiamen University, Xiamen 361102, China; leyloria@gmail.com; 2 School of Public Affairs, Xiamen University, Xiamen 361005, China; 3 School of Public Health, Xiamen University, Xiamen 361102, China; shaw.1995@hotmail.com; 4 Key Laboratory of Health Technology Assessment of Fujian Province, School of Public Health, Xiamen University, Xiamen 361102, China

**Keywords:** social engagement trajectory, heterogeneity, aging, sex differences

## Abstract

This study aimed to identify the heterogeneous trajectory classes of social engagement among older adults in China and examine sex differences to determine how sociodemographic characteristics and health status impact these trajectory classes. A sample of 8117 participants aged ≥65 years from the Chinese Longitudinal Healthy Longevity Survey was included. Growth mixture modeling was conducted to estimate the trajectory classes of social engagement. Logistic regression was adopted to analyze the associations between individual characteristics and trajectory classes. Three trajectory classes of social engagement were identified: the high-decline (35.3%), medium-decline (57.6%), and low-increase (7.1%). Men were less likely to be in the medium-decline and low-increase classes than women. Older men benefited from economic factors more than women. Education exhibited a stronger protective effect on the high-decline class for older women. High physical functioning might enable older adults with the lowest initial level of social engagement to make efforts to engage in social activities, which was stronger for older men than women. For both men and women, a proper cognitive state and positive emotions were in favor of social engagement. These findings are valuable for achieving sex equity in social engagement development for healthy and active aging.

## 1. Introduction

Decreasing fertility rates and increasing longevity contribute to the continued aging of the population globally. Socioeconomic development, especially in developing countries, has often lagged behind the fast speed of aging in populations [1]. As the largest developing country, China has one of the most rapidly aging populations, undergoing huge demographic and health transitions. The number of older Chinese people aged ≥65 were 172 million (12.0%) in 2017 and is projected to reach 307 million in 2050 [2]. There is an increasing concern on the health issues of older Chinese people, including comorbidities, loneliness, and physical and cognitive impairments [3,4]. However, more than physical and psychological conditions, older Chinese faced considerable social problems. With the implementing of one-child policy and the development of urbanization, nearly half older Chinese people now become ”empty nesters”, and the loss of intimate relationships with great changes in physical health status might cause loneliness and social isolation among these older people [4]. Given the diversity of health issues experienced by older people, there is a growing recognition of the need to assess the heterogeneity of physical, psychological, and social health [5,6,7]. The concept of ”active aging” has been proposed as a policy framework and global goal by the World Health Organization to enhance the well-being of older adults and address the serious health challenges of aging. Given the rapidly aging population and marked health heterogeneity, and the lagging behind of aging programs [8], there is an urgent need to promote ”active aging” among older people in China [1].

Social engagement (SE) is defined as an individual’s social behavior and social structure and is a significant component of active aging, a central topic in many aging studies [9]. When assessing the SE of the older population, much research has focused on SE’s inactive status. Several recent studies have provided some insights into the changing patterns of SE based on the dynamic perspective of aging [10,11]. These studies have examined the relationship between SE and personal characteristics based on the assumption that the older population is homogeneous. Concrete evidence across different countries indicates that sociodemographic characteristics, such as sex, age, education, residence status, occupation, and pensions, play essential roles in the SE among older adults [12,13,14], and that their SE levels are related to disability, cognitive impairment, loneliness, and general well-being [15,16,17].

Under the assumption of population-based homogeneity, disengagement theory argues that older people withdraw from society and social activities during the inevitable process of aging [18]. Socioemotional selectivity theory also points out the functional declines in social connections throughout adulthood [19]. Given that, older adults might follow a normative and ubiquitous SE trajectory with gradual deterioration. However, the critics of disengagement theory lay emphasis on the diversity in aging patterns, including the sex differences [20]. Older people might not experience the same patterns of SE changes. Instead, the potential heterogeneity of SE trajectories that exist between individuals can be represented as possible subclasses with similar characteristics in SE trajectories. Achenbaum and Bengtson already pointed out that the heterogeneity among the older population was not given sufficient prominence [20]. It is crucial and necessary to identify the heterogeneous trajectory classes of SE for the largest old population in the world to promote an active aging process and address the population-based heterogeneity.

Substantial evidence consistently suggests that sex has a significant effect on the level of SE among older adults, with some studies indicating that women benefit more from SE than men [21]. In contrast, some other studies have reported inconsistent conclusions, indicating that SE is more effective for men [22]. According to the socioemotional selectivity theory [19], women and men might present different social interactions based on identity maintenance or emotional goals. When time is perceived as limited, those goals might change and show sex difference among older adults. Women tend to have more social support and involve themselves in social networks, consisting of family, friends, and relatives. By contrast, men have more social connections in work environments but tend to be mainly supported by their spouses after retirement [23]. It is well-established that physical and psychological health is an outcome and driver of SE. One study in northern Europe suggested that both men and women in need of help had limited diversity of social relations compared to well-functioning individuals [24]. An American study showed that physical and cognitive limitations affect subsequent SE among older men, while older women might attempt to pursue SE even in the face of these limitations [21]. Because of these differences in additional sociodemographic and health characteristics, older men and women might have not only different SE statuses but also experience different SE trajectories. Nevertheless, discussions on how other sociodemographic characteristics and health status impact the sex differences of SE trajectories, especially for heterogeneous trajectory classes, remain limited.

This study aimed to assess the heterogeneity of SE trajectory among older Chinese citizens and examine the sex differences in terms of the associations between these heterogeneous trajectories and individual sociodemographic characteristics and health status.

## 2. Materials and Methods

In this section, we introduce the data source, variables measurement, and analytic strategy of our study.

### 2.1. Data Source and Study Population

The study’s data were provided by the Chinese Longitudinal Healthy Longevity Survey (CLHLS) [25]. It was the first nationwide longitudinal survey on health and relevant factors among the older population in China. The CLHLS covers 22 of the 31 mainland provinces, encompassing 85% of the total population in China. The CLHLS conducted eight investigations (1998, 2000, 2002, 2005, 2008–2009, 2011–2012, 2014, and 2018). The CLHLS was approved by the research ethics committees of Duke University and Peking University (IRB00001052–13074), and are publicly available at the Peking University Open Research Data (http://opendata.pku.edu.cn/dataverse/CHADS). The dataset is publicly accessible to scholars for nonprofit purposes. More details of the study design have been described in previous studies, and the survey data have been widely reported as high-quality [26].

This study used six recent investigations of the CLHLS. The first two were excluded because they mainly targeted people aged ≥80 years. The CLHLS conducted interviews with 16,064 individuals in 2002, selected as the baseline wave of investigation in this study. For trajectory analysis, those who had complete information on critical variables for at least two waves were included. Thus, the study population consisted of 8117 participants aged ≥65 years. Table S1 (see Supplementary Materials) presents the distribution of missing observations for SE in each wave and baseline variables. The baseline characteristics of the analytic sample and those excluded were compared by univariable and multivariable logistic regression, as reported in Table S2 (see Supplementary Materials). The results of logistic regression showed that the analytic sample was more likely to report higher SE at baseline.

### 2.2. Social Engagement

The main outcome of interest was a time-varying continuous variable of SE during the six waves of investigation in 16 years. According to previous studies [11,27,28,29], SE was assessed by five dichotomous indicators: marital status (0 = unmarried, 1 = married); living arrangement (0 = living alone, 1 = living with family, friends, or other persons); the availability of help when required (0 = nobody, 1 = somebody), the availability of a confidant (0 = nobody, 1 = somebody), and participation in social activities (0 = no, 1 = yes). A total SE score was formed by summing up the five indicators for each wave, and a higher score indicated greater SE.

### 2.3. Covariates

All the covariates were measured during the first wave in this study, which was divided into two subcategories: sociodemographic characteristics and health status.

The sociodemographic characteristics consisted of age (65–74, 75–84, or 85+ years), sex (man or woman), ethnicity (Han ancestry or minority), education (0, 1–5, 6–9, or 10+ years), occupational status (high or low level), type of residence (urban or rural), region (eastern, central, or western), sufficient financial support (yes or no), and having a pension (yes or no). Occupational status was defined as a high level if the participant’s primary occupation before age 60 was professional, technical, governmental, institutional, managerial, or military personnel, similar to previous CLHLS studies [30]. The regional divisions for the 22 sampled provinces in CLHLS conformed to the classification of the National Bureau of Statistics of China, mainly based on sociodemographic development levels and geography.

Health status consisted of physical and psychological health dimensions. Two health indicators in the physical dimension were measured: activities of daily living (ADL) and chronic condition. Using the Katz activities of daily living scale, the ADL index constituted a total score, ranging from 0 to 6, with lower scores suggesting higher physical functioning. The chronic condition was assessed by the total number of chronic diseases, including hypertension, diabetes, cardiac disease, cerebrovascular disease, bronchitis/emphysema/asthma/pneumonia, tuberculosis, cancer, gastric/duodenal ulcer, Parkinson’s disease, arthritis, and dementia. Indicators of psychological health dimensions consisted of cognitive function and positive emotions. Cognitive function was measured by the mini-mental state examination (MMSE). The range of the MMSE score was 0–30, with higher scores indicating better cognitive function. The measure of positive emotions was defined by asking the participants whether they felt anxious, isolated, and useless as an elderly person (0 = always, 1 = often, 2 = sometimes, 3 = seldom, or 4 = never). Thus, the positive emotions index ranged from 0 to 12, with a higher score suggesting better emotional condition. The multiple imputation approach was applied to reduce the influence of missing values on these covariates in the analyses.

### 2.4. Statistical Analysis

Sex differences in sociodemographic characteristics and health status were compared by the chi-squared test for categorical variables and by nonparametric Wilcoxon rank–sum test for non-normal continuous variables.

Growth mixture modeling (GMM) was implemented to capture the heterogeneity of SE trajectory. This approach clusters individuals into latent classes based on their growth trajectories and accommodates missing values [31,32]. Latent trajectory classes and interindividual variations within each class specify the differences between individuals [33]. Based on previous recommendations [31,34], latent growth curve modeling (LGCM) and latent class growth modeling (LCGM) should be conducted initially to explore the growth curve shape and the number of latent trajectory classes before GMM. LGCM was used to estimate the mean growth trajectory and interindividual variations. LCGM constitutes a cluster analytic extension of LGCM, which estimates the mean trajectory for each class without interindividual variations. LGCM and LCGM can be regarded as special cases of GMM [32]. The best class solutions of LCGM and GMM are obtained by increasing the number of latent classes until no improvements are observed in the model performance. GMMs are fitted after LCGMs to determine interindividual variations within each class [34]. The performance of LCGMs and GMMs was compared based on statistical indices and interpretability. Statistical indices consisted of sample-size adjusted Bayesian information criterion (SABIC), entropy, bootstrap likelihood ratio test (BLRT), and Vuong–Lo–Mendell–Rubin likelihood ratio test (VLMR-LRT) [34]. SABIC belongs to an information criterion with a more significant reduction in SABIC, representing an improvement of the model fit. Entropy is a measure of classification precision, which ranges from 0 to 1, with higher values indicating better class separation. The VLMR-LRT and BLRT compare a k-1-class model with a k-class model, where a significant *p*-value suggests that the k-class model provides a better fit than the k-1-class model. In addition, it was essential for each class in the model to contain sufficient individuals, no less than 5% of the sample. The interpretability of each trajectory class was also considered to select the final model.

Finally, a multinomial logistic regression model was adopted to assess sex differences of SE trajectory classes and the impact of health status and other sociodemographic characteristics. Statistical analyses were performed with Mplus 7.4 (Muthén and Muthén, 2015) and R version 4.0.0 (R Core Team, Vienna, Austria, 2020). A *p*-value of <0.05 was regarded as statistically significant.

### 2.5. Sensitivity Analysis

The sample was changed from those with complete data for at least two waves into three waves (*n* = 4159), and trajectory class analysis performed among this sample to verify the robustness of the results in trajectory analysis. Moreover, according to the definition of SE in this study, married people were considered more likely to live with their spouse, ask them for help, and take their spouse as a confidant than unmarried ones. Thus, we further excluded people with a spouse as cohabitant or confidant, and performed trajectory class analysis for the rest (*n* = 5160).

## 3. Results

In this section, we describe baseline characteristics of the sample and present the heterogeneous classes of SE and their associations with sociodemographic characteristics and health status. Sensitivity analysis results are also provided.

### 3.1. Baseline Characteristics of Older Adults

Table 1 presents the baseline characteristics of older adults and their sex differences. This study consisted of 3669 men and 4448 women. The mean SE level of older adults was 3.4, with men exhibiting a higher level in all the five indicators of SE (all *p* < 0.05). Significant differences were found between men and women in age, ethnicity, occupational status, education, region, sufficient financial support, pension, and the four measures of health status (all *p* < 0.001).

### 3.2. Heterogeneous Trajectory Classes of Social Engagement

A linear mean trajectory was found with a negative slope for the SE of older adults (intercept = 3.37, *p* < 0.001; slope = –0.07, *p* < 0.001). Table 2 presents the performance of LCGM with 2–5 classes. A notable reduction in SABIC was observed between the 2-class and 3-class LCGMs, suggesting a better fit of the latter. Although the results of VLMR-LRT and BLRT indicated that models with more classes were more favorable, the 3-class LCGM exhibited higher entropy (0.81) than the 4-class and 5-class models (0.80 and 0.76, respectively), and its smallest class was >5%, indicating that the 3-class solution was preferable. Then, the variances of intercept and slope were freely estimated in GMM, and the GMM with three classes was adopted as the final model (SABIC = 58,180.5, entropy = 0.78, VLMR-LRT *p* < 0.001, BLRT *p* < 0.001).

**Figure 1:**
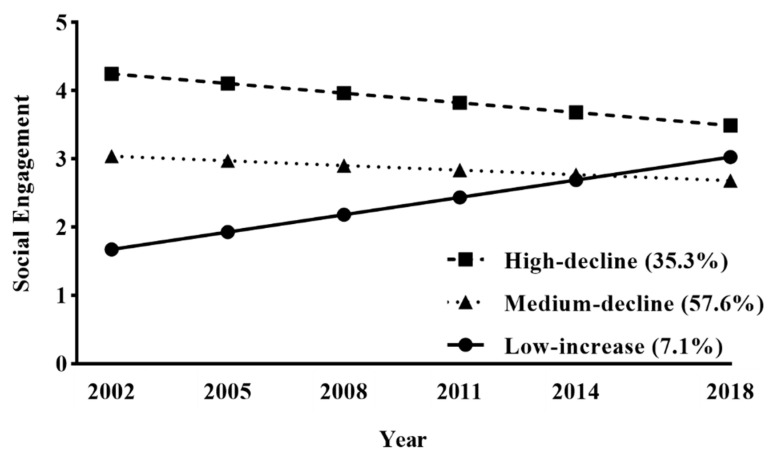
Heterogeneous trajectory classes of social engagement.

Figure 1 presents three classes of SE trajectory. The first class, representing 35.3% of the sample, had a high SE level initially, which decreased slightly later (intercept = 4.25, *p* < 0.001; slope = –0.14, *p* < 0.001) and was labeled as high-decline (HD). The second class consisted of the most participants (57.6%) whose initial SE levels were medium, which decreased slowly afterward (intercept = 3.04, *p* < 0.001; slope = –0.07, *p* < 0.001) and named medium-decline (MD). The last SE trajectory exhibited the lowest initial level and then increased rapidly (intercept = 1.67, *p* < 0.001; slope = 0.25, *p* < 0.001) during the subsequent years. Thus, this class was referred to as low-increase (LI) and consisted of 7.1% of participants.

### 3.3. Associations of Sociodemographic Characteristics and Health Status with Social Engagement Trajectory Classes

Table 3 presents the results of univariable and multivariable logistic regression on the associations between baseline characteristics and latent trajectory class membership of SE. The HD class was regarded as the reference class since it represented a relative higher level of SE than others during the study period. According to the results of the univariable analysis, the MD class was significantly different from the HD class in baseline characteristics except for residence and living in the central (vs. western) region. No significant difference was found in ethnicity and the central (vs. western) region between the LI and HD classes. The results of the multivariable analysis showed that women, older age, and no-pension variables were risk factors to the MD and LI classes. The minorities and those residing in urban areas were significantly more likely to be classified in the MD class than the HD class. Those from the western region of China (vs. the eastern region) and with sufficient financial support had protective effects on the HD class than the LI class. Concerning health status, participants with higher cognitive functioning and more positive thoughts had positive association with the HD class. Subjects with higher physical functioning were more likely to be in the LI class than HD class.

Table 4 presents sex differences in the associations between baseline characteristics and SE trajectory class membership. It was found among men that those who were of Han ancestry (OR = 0.43, *p* < 0.001), had high occupational status (OR = 0.75, *p* < 0.05) and resided in urban areas (OR = 1.29, *p* < 0.01) were only associated with the MD (vs. HD) class. Men with >10 years of education (vs. no education) were at more risk of being classified in the LI (vs. HD) class (OR = 2.47, *p* < 0.05). Educated women with >10 years of education, compared to the uneducated, were protected from the MD (vs. HD) class (OR = 0.48, *p* < 0.05). Both education of 1–5 years (OR = 0.58, *p* < 0.05) and 6–9 years (OR = 0.35, *p* < 0.05) for women were negatively associated with membership in the LI class. In terms of health status, the significant associations of SE trajectory classes with ADL, MMSE score, and positive emotions remained unchanged.

### 3.4. Robustness of Trajectory Classification

Three trajectory classes were identified among subjects who had complete information on SE for at least three waves (see Supplementary Figure S1). The shape and proportion of SE trajectories were similar to the current analytic sample. After excluding the spouse as a cohabitant or confidant, two classes of SE trajectory were identified (see Supplementary Figure S2). The HD class of SE trajectory found by the current analytic sample was not identified here, while there were still another two classes of change patterns (declining and increasing) of SE.

## 4. Discussion

Based on nationally representative data of the older population in China, the present study evaluated the heterogeneity of the growth trajectories of SE by identifying three latent classes: the HD, MD, and LI. These classes had characteristics similar to previous SE trajectory classifications among older Americans [21,35]. Remarkably, the LI class exhibited a noticeable trend of continuous increment, which was the exact opposite of the mean trajectory of the population. Thus, the use of the homogeneously decreasing trajectory of SE to describe older adults might be misleading. Subsequent analyses of the characteristics of sociodemographic data and health status indicated that the heterogeneous trajectory classes provided a clear distinction of SE development patterns, building a valid base to compare sex differences. The findings of this study revealed that introducing heterogeneity provided a more comprehensive assessment of complex development patterns of SE among the largest older population in the world, advancing previous studies [28,36].

The results showed that the mean SE trajectory of older adults tended to deteriorate slowly over time, consistent with a previous study [37]. Regarding the aging process, older adults might potentially lose their social roles, leading to the loss of social bonds [38]. Most older adults, including those in the HD and MD classes, followed a declining change pattern of SE trajectory regardless of their initial SE level. Given cultural traditions and financial reasons, older Chinese people devote most of their retirement time to caring for their grandchildren and barely have any time for other forms of socialization [3]. Moreover, the types of social activities for older adults in China are limited and mainly consist of static entertainments, such as playing mah-jong or cards. These activities might lead to increased passive and sedentary behaviors, which may further reduce the SE level in older adults [39]; however, static activities might be an effective way to promote SE for older adults with limited physical mobility. As it is well-established that SE plays a vital role in the positive outcomes of older adults [15], promoting the SE of older individuals should be one of the major priorities in the development of aging agenda for the Chinese government. Specifically, health policymakers should take the heterogeneity of older people into account to improve their SE.

Remarkably, significant sex differences exist among SE trajectory classes for older Chinese people; however, they have not yet been highlighted. The results of the present study suggest that men are less likely than women to be in the MD and LI classes. Older men have higher educational levels and income in addition to more employment opportunities than older women in China, generally leading to a higher status and more economic prosperity. Thus, older men of higher social and family status typically have more resources and capital at their disposal to exchange in society [40]. Compared to men, older women in China are more seriously disadvantaged [41]. They are older, more likely to live alone as widows, and rely economically on their spouses or children. Moreover, with much more time spent on childcare than men [42], their social contact is usually limited to their families, leading to lower levels of SE. Sex differences of SE trajectory classes in this study might be attributed to other sociodemographic characteristics and physical and psychological health status of each sex.

Concerning age, the results of the present study showed that compared to the youngest group, the older group who had a higher likelihood of being unmarried (see Supplementary Table S3) tended to have lower initial SE levels; however, those with the lowest level initially tended to increase in subsequent years. The impact of age on SE trajectory class was stronger among women than men. With the decline of childcare-related time and activities, older women are more likely than men to develop friendships and establish new contacts with a variety of people in the neighborhood and community. On the contrary, older men are more likely to retain a pool of social contacts built earlier before retirement [43]. During the aging process, older men might experience a stage when their old contacts vastly diminish, whereas no new bonds are connected, especially for a widower, necessitating interventions to minimize social isolation for older men.

In addition, there was a more distinct association between education and SE trajectory classes after sex stratification. Education is a strong predictor of longevity [30], which might cause a low baseline level of SE for older men with an education of >10 years. With higher education, however, they might acquire a greater ability to improve their SE levels in later years [44]. Concerning women, most of them were illiterate in China; thus, education is especially crucial to protect women against adverse health conditions, such as low SE levels. However, enrolling in universities has become increasingly difficult for older individuals in China as one crucial way of acquiring SE. Besides, the essential services of the majority of current communities for seniors and old-age care institutions are far from meeting the multifaceted demands of the elderly. These problems reveal the severe lagging of aging conditions behind the economic and social development of China. It is essential to provide better education and more SE opportunities, especially for older women.

Compared with older adults from eastern regions, the influence of the western regions on the LI class among the whole sample became insignificant after sex stratification. In contrast, men from the central regions were significantly associated with increased likelihood to be in the MD class. In China, the central and western regions have relatively poor economic conditions [45]; older adults from these regions might have lower SE levels than those from the eastern regions. However, in the cities of western provinces, such as Sichuan and Chongqing, older individuals generally enjoy a casual lifestyle and partake in social activities, such as playing mah-jong, which takes up most of their time, possibly offsetting the adverse effects of poor economic condition on SE. Existing evidence suggests that men from the central regions experience various difficulties in marriage [46]. Their marriage may be subject to a higher risk of instability, adversely affecting SE. Regional disparities in the SE trajectories of both older men and women in China necessitate further studies.

Good economic conditions and social welfare also had protective effects on SE trajectories, consistent with previous studies [47]. Sufficient financial support might encourage both men and women to engage in social activities and retain social contacts. The positive effects of economic factors on SE were evident among men rather than women. Older men with pensions were less likely to have an initially low level of SE. Thus, more attention must be paid to the possible changes of SE in older men with the worsening of the current economic conditions.

In terms of health status, physical functioning of older people only distinguished the LI and HD classes, whereas chronic condition was not significantly associated with SE trajectories. Higher physical functioning might enable older adults with the lowest SE level to make efforts to engage in social activities. This effect was stronger in older men than women. However, the available studies have pointed out that the significant factor affecting the engagement of older individuals in society is not disease or physical functioning; instead, it is their psychological health [48]. Because of the ”one-child policy” and a large number of migrant laborers, the proportion of ”empty nest” families, especially in rural areas, is growing, posing potential psychological problems and leading to reduced SE in older individuals in China. The results showed that a better cognitive state and more positive emotions were in favor of SE. Women benefit slightly more from proper cognitive functioning than men, possibly because women tend to live longer and have a higher risk of cognitive decline. Older women with a poor cognitive state were more likely to have difficulty with poor social networks [49].

Moreover, the results suggested that positive emotions had a slightly stronger influence on men than women. Compared to the emotional coping styles of women, men tend to use rational coping styles [50]; hence, men might experience more significant improvements in SE as a result of positive emotions. Compared to earlier studies [51,52], the findings of this study enhanced the understanding of sex disparities in the heterogeneous SE trajectory classes for older adults.

There were some limitations in the present study that should be considered. Firstly, given the half attrition rate, the analytic sample in the current study might represent a positive state of health among older Chinese people. Although the results of sensitivity analysis suggested that the trajectory classes were robust, it is still of note that the current results should be extrapolated cautiously. Secondly, according to the definition of SE in this study, married couples might take their spouse as cohabitant or confidant, which might lead to a measurement bias of SE. The sensitivity analysis provided evidence that married individuals who are highly dependent on their spouses tend to have higher SE levels, but this might not disturb their changing patterns of SE trajectory. The measure of SE in this study might be uncommon in social science, but it is usually used in CLHLS studies. Thirdly, factors such as health status were measured using baseline data, which might change in the subsequent years and impact the SE trajectories. Physical and psychological health might interact with SE levels in each wave, as physical functioning might be a foundation to engage social activities whereas stable SE level might encourage people to think positively. It would be fruitful to evaluate, in subsequent studies, the potential mechanism and changes existing in these interactions. Finally, this study chose wave, rather than age, as time to assess the population-based heterogeneity. An age-based model may be used to improve the understanding about the effect of age on SE trajectory in further study.

## 5. Conclusions

In conclusion, we identified three classes of heterogeneous SE trajectory associated with different sociodemographic characteristics for older adults in China. These heterogeneous trajectories showed abundant gender disparities and had different characteristics of other sociodemographic information between men and women. Introducing heterogeneity provided a more comprehensive assessment of complex growth patterns of SE for older adults in China. Moreover, compared with earlier studies, the findings of this study strengthened the understanding of sex differences in the SE trajectory classes for older adults, and could help identifying high-risk groups for both older men and older women. This study provides important evidences for achieving equity between the sexes in SE development to accomplish the goal of healthy and active aging in older population.

## Figures and Tables

**Table 1:** Baseline characteristics of older adults and their sex differences.

Variables	*Total (* *n = 8117)*	*Man (* *n = 3669)*	*Woman (* *n = 4448)*	*p-Value* ^1^
	% or Mean (SD)	% or Mean (SD)	% or Mean (SD)	
Social engagement (0–5)	3.4 (1.0)	3.7 (1.0)	3.1 (0.9)	<0.001
Marital status (ref: Unmarried)				
Married	39.5	57.1	24.9	<0.001
Living arrangement (ref: Alone)				
With people	86.1	89.1	83.7	<0.001
Available of help (ref: No)				
Yes	97.0	97.2	96.8	<0.050
Available of confidant (ref: No)				
Yes	93.8	94.7	93.0	<0.001
Social activities (ref: No)				
Yes	20.9	27.9	15.0	<0.001
Sociodemographic characteristics				
Age (ref: 65–74)				
75–84	29.6	32.9	26.8	<0.001
85+	39.3	31.9	45.4	
Ethnicity (ref: Minority)				
Han	93.6	94.4	93.0	<0.001
Occupational status (ref: Low level)				
High level	10.6	18.8	3.8	<0.001
Education years (ref: 0)				
1–5	25.8	40.3	13.8	<0.001
6–9	10.7	18.4	4.3	
10+	5.7	10.2	2.1	
Residence (ref: Rural)				
Urban	43.9	43.7	44.1	0.52
Region (ref: Eastern China)				
Central China	25.2	24.4	25.9	<0.001
Western China	44.6	45.8	43.6	
Sufficient financial support (ref: No)				
Yes	80.6	82.8	78.8	<0.001
Pension (ref: No)				
Yes	22.0	34.4	11.8	<0.001
Health status				
ADL (0–5)	0.3 (1.0)	0.2 (0.8)	0.4 (1.1)	<0.001
Chronic disease numbers (0–11)	0.5 (0.8)	0.5 (0.8)	0.5 (0.7)	<0.001
MMSE score (0–30)	24.7 (6.9)	26.3 (5.7)	23.4 (7.4)	<0.001
Positive emotions (0–12)	8.3 (2.4)	8.8 (2.3)	8.0 (2.4)	<0.001

Note: SD = standard deviation; MMSE = mini-mental state examination; ADL = activities of daily living; ref = reference. ^1^ *p* values based on chi-squared tests (for categorical variables), and nonparametric Wilcoxon rank–sum tests (for continuous variables).

**Table 2:** Performance of latent class growth models and growth mixture models.

Model	Indices				
	SABIC	Entropy	VLMR-LRT *p*-Value	BLRT *p*-Value	Smallest Class
LCGM					
2-class LCGM	60,243.5	0.77	<0.001	<0.001	36%
3-class LCGM	58,701.3	0.81	<0.001	<0.001	8%
4-class LCGM	58,245.0	0.80	<0.001	<0.001	5%
5-class LCGM	57,974.7	0.76	0.001	<0.001	2%
GMM					
3-class GMM	58,180.5	0.78	<0.001	<0.001	7%

Note: SABIC = sample-size adjusted Bayesian information criteria; VLMR-LRT = Vuong–Lo–Mendell–Rubin likelihood ratio test; BLRT = bootstrapped likelihood ratio test; LCGM = latent class growth modeling; GMM = growth mixture modeling.

**Table 3:** Associations of baseline characteristics with social engagement trajectory classes among older adults by univariable and multivariable logistic regression.

Variables	MD vs. HD (*n* = 7544)		LI vs. HD (*n* = 3443)	
	Crude OR (95%CI)	Adjusted OR (95%CI)	Crude OR (95%CI)	Adjusted OR (95%CI)
Sociodemographic characteristics				
Sex (ref: Woman)				
Man	0.25 (0.23–0.28) ***	0.32 (0.27–0.36) ***	0.23 (0.19–0.28) ***	0.30 (0.23–0.38) ***
Age (ref: 65–74)				
75–84	2.75 (2.44–3.10) ***	2.87 (2.51–3.27) ***	2.49 (1.96–3.15) ***	2.39 (1.84–3.09) ***
85+	12.31 (10.75–14.09) ***	10.02 (8.56–11.74) ***	7.64 (6.02–9.69) ***	6.66 (4.99–8.87) ***
Ethnicity (ref: Minority)				
Han	0.54 (0.44–0.67) ***	0.55 (0.42–0.71) ***	0.97 (0.63–1.49)	1.20 (0.72–2.01)
Occupational status (ref: Low level)				
High level	0.30 (0.26–0.35) ***	0.81 (0.65–1.02)	0.22 (0.15–0.32) ***	0.59 (0.34–1.01)
Education years (ref: 0)				
1–5	0.38 (0.34–0.42) ***	0.89 (0.77–1.03)	0.32 (0.26–0.40) ***	0.79 (0.60–1.04)
6–9	0.25 (0.22–0.29) ***	1.00 (0.82–1.22)	0.19 (0.13–0.27) ***	0.96 (0.62–1.48)
10+	0.22 (0.18–0.27) ***	1.00 (0.74–1.34)	0.24 (0.15–0.37) ***	1.75 (0.95–3.25)
Residence (ref: Rural)				
Urban	0.93 (0.85–1.02)	1.27 (1.11–1.44) ***	0.70 (0.58–0.84) ***	1.21 (0.95–1.54)
Region (ref: Eastern China)				
Central China	1.00 (0.88–1.14)	1.11 (0.95–1.30)	1.00 (0.79–1.26)	0.87 (0.65–1.15)
Western China	0.83 (0.74–0.92) ***	0.93 (0.81–1.07)	0.70 (0.57–0.87) **	0.77 (0.60–0.99) ^*^
Sufficient financial support (ref: No)				
Yes	0.72 (0.64–0.82) ***	0.94 (0.81–1.09)	0.33 (0.27–0.41) ***	0.54 (0.42–0.68) ***
Pension (ref: No)				
Yes	0.30 (0.27–0.33) ***	0.53 (0.45–0.62) ***	0.23 (0.18–0.30) ***	0.46 (0.32–0.65) ***
Health status				
ADL (0–6)	1.67 (1.54–1.81) ***	1.04 (0.96–1.13)	1.28 (1.15–1.42) ***	0.80 (0.70–0.93) **
Chronic disease numbers (0–11)	0.88 (0.83–0.94) ***	1.02 (0.94–1.10)	0.98 (0.87–1.10)	1.13 (0.98–1.30)
MMSE score (0–30)	0.87 (0.86–0.88) ***	0.97 (0.95–0.98) ***	0.89 (0.87–0.90) ***	0.95 (0.93–0.97) ***
Positive emotions (0–12)	0.85 (0.83–0.87) ***	0.91 (0.89–0.94) ***	0.75 (0.72–0.78) ***	0.84 (0.80–0.88) ***

Note: OR = odds ratio; CI = confidence interval; MD = “medium-decline”; HD = “high-decline”; LI = “low-increase”; MMSE = mini-mental state examination; ADL = activities of daily living; ref = reference; * *p* < 0.05, ** *p* < 0.01, *** *p* < 0.001.

**Table 4:** Sex differences in the associations between baseline characteristics and social engagement trajectory classes among older adults by univariable and multivariable logistic regression.

Variables	Man (*n* = 3669)				Woman (*n* = 4448)			
	MD vs. HD		LI vs. HD		MD vs. HD		LI vs. HD	
	Crude OR (95%CI)	Adjusted OR (95%CI)	Crude OR (95%CI)	Adjusted OR (95%CI)	Crude OR (95%CI)	Adjusted OR (95%CI)	Crude OR (95%CI)	Adjusted OR (95%CI)
Sociodemographic characteristics								
Age (ref: 65–74)								
75–84	3.23 (2.69–3.86) ***	3.17 (2.63–3.83) ***	2.15 (1.46–3.17) ***	2.07 (1.38–3.10) ***	2.84 (2.38–3.39) ***	2.52 (2.09–3.03) ***	3.04 (2.22–4.14) ***	2.55 (1.83–3.55) ***
85+	10.05 (8.31–12.16)	8.81 (7.15–10.85) ***	4.74 (3.21–6.99) ***	4.02 (2.60–6.22) ***	20.14 (15.80–25.65) ***	13.69 (10.45–17.94) ***	14.22 (10.10–20.00) ***	10.20 (6.85–15.18) ***
Ethnicity (ref: Minority)								
Han	0.51 (0.38–0.69) ***	0.43 (0.30–0.61) ***	0.81 (0.40–1.65)	1.02 (0.46–2.24)	0.63 (0.46–0.86) **	0.73 (0.51–1.05)	1.18 (0.67–2.08)	1.37 (0.70–2.69)
Occupational status (ref: Low level)								
High level	0.45 (0.38–0.54) ***	0.75 (0.58–0.96) *	0.34 (0.21–0.56) ***	0.70 (0.37–1.33)	0.44 (0.32–0.61) ***	1.24 (0.75–2.04)	0.32 (0.16–0.66) **	0.51 (0.17–1.57)
Education years (ref: 0)								
1–5	0.66 (0.56–0.78) ***	0.89 (0.75–1.07)	0.60 (0.42–0.85) **	0.99 (0.67–1.46)	0.53 (0.44–0.65) ***	0.90 (0.72–1.14)	0.44 (0.31–0.64) ***	0.58 (0.38–0.90) *
6–9	0.48 (0.40–0.59) ***	1.05 (0.83–1.34)	0.45 (0.29–0.72) ***	1.38 (0.81–2.35)	0.32 (0.24–0.43) ***	0.74 (0.51–1.09)	0.14 (0.06–0.33) ***	0.35 (0.14–0.89) *
10+	0.42 (0.33–0.54) ***	1.16 (0.83–1.61)	0.47 (0.27–0.82) **	2.47 (1.18–5.15) *	0.33 (0.21–0.51) ***	0.48 (0.25–0.93) *	0.37(0.16–0.84) *	0.89 (0.26–3.08)
Residence (ref: Rural)								
Urban	0.84 (0.73–0.96) **	1.29 (1.08–1.54) **	0.57 (0.41–0.78) ***	1.14 (0.77–1.69)	1.03 (0.89–1.19)	1.26 (1.04–1.53) *	0.80 (0.63–1.02)	1.30 (0.94–1.79)
Region (ref: Eastern China)								
Central China	1.06 (0.89–1.27)	1.29 (1.05–1.60) *	0.68 (0.45–1.03)	0.77 (0.48–1.22)	0.94 (0.77–1.15)	0.93 (0.73–1.17)	1.12 (0.82–1.52)	0.87 (0.60–1.28)
Western China	0.86 (0.73–1.00)	0.99 (0.82–1.20)	0.70 (0.50–0.99) *	0.76 (0.52–1.12)	0.80 (0.68–0.95) *	0.87 (0.70–1.07)	0.69 (0.52–0.92) *	0.78 (0.55–1.10)
Sufficient financial support (ref: No)								
Yes	0.75 (0.62–0.89) ^**^	1.00 (0.81–1.24)	0.34 (0.24–0.47) ^***^	0.57 (0.39–0.83) **	0.78 (0.64–0.94) **	0.88 (0.70–1.09)	0.36 (0.28–0.47) ***	0.51 (0.36–0.70) ^***^
Pension (ref: No)								
Yes	0.46 (0.40–0.53) ***	0.51 (0.42–0.63) ***	0.27 (0.18–0.40) ***	0.32 (0.19–0.55) ***	0.34 (0.28–0.41) ***	0.60 (0.45–0.79) ***	0.35 (0.24–0.51) ***	0.79 (0.48–1.30)
Health status								
ADL (0–6)	1.37 (1.24–1.52) ***	1.03 (0.92–1.15)	1.04 (0.82–1.32)	0.75 (0.56–0.99) *	1.83 (1.59–2.10) ***	1.03 (0.90–1.18)	1.36 (1.17–1.57) ***	0.79 (0.65–0.95) *
Chronic disease numbers (0–11)	0.86 (0.79–0.95) **	0.97 (0.88–1.08)	0.94 (0.77–1.15)	1.05 (0.84–1.31)	0.93 (0.84–1.02)	1.08 (0.96–1.22)	1.04 (0.89–1.21)	1.18 (0.98–1.43)
MMSE score (0–30)	0.91 (0.90–0.92) ***	0.98 (0.96–0.99) **	0.93 (0.91–0.95) ***	0.97 (0.94–1.00) *	0.86 (0.85–0.88) ***	0.95 (0.94–0.97) ***	0.86 (0.84–0.88) ***	0.93 (0.91–0.96) ***
Positive emotions (0–12)	0.87 (0.84–0.90) ***	0.91 (0.87–0.94) ***	0.76 (0.71–0.82) ***	0.81 (0.75–0.87) ***	0.88 (0.85–0.91) ***	0.92 (0.88–0.95) ***	0.78 (0.74–0.83) ***	0.84 (0.79–0.90) ***

Note: OR = odds ratios; CI = confidence interval. MD = “medium-decline”; HD = “high-decline”; LI = “low-increase”; MMSE = mini-mental state examination; ADL = activities of daily living; ref = reference. * *p* < 0.05, ** *p* < 0.01, *** *p* < 0.001.

## Supplementary Materials

The following are available online at https://www.mdpi.com/1660-4601/17/22/8322/s1, Figure S1: Heterogeneous trajectory classes of social engagement for older adults with complete information for at least three waves (*n* = 4159), Figure S2: Heterogeneous trajectory classes of social engagement for older adults with different definition of social engagement by excluding spouse from cohabitant or confidant (*n* = 5160), Table S1: Missing observations for social engagement in each wave and baseline variables, Table S2: Comparison of baseline variables between analytical sample and drop-out sample, Table S3: Marital status of older adults between three age groups.
