# Supplementary material for: Heterogeneous Trajectory Classes of Social Engagement and Sex Differences for Older Adults in China
Source: Int J Environ Res Public Health. 2020 Nov 11;17(22):8322. doi: 10.3390/ijerph17228322 (PMC7696123; doi:10.3390/ijerph17228322)
Supplement: Supplementary file 1 [file ijerph-17-08322-s001.pdf]

Supplementary material

Supplementary Table 1. Missing Observations for Social Engagement in Each Wave and Baseline Variables.

**Variables.**	**n**	**%**
**Social engagement**		
Wave 1	2	0.0
Wave 2	7913	49.3
Wave 3	11875	73.9
Wave 4	13649	85.0
Wave 5	14500	90.3
Wave 6	15347	95.5
**Sociodemographic characteristics**		
Age	0	-
Sex	0	-
Ethnicity	0	-
Occupational status	0	-
Education years	105	0.7
Residence	0	
Region	0	
Sufficient financial support	5	
Pension	13	0.1
**Health status**		
ADL (0-6)	50	0.3
Chronic diseases numbers (0-11)	1646	10.2
MMSE score (0-30)	9	0.1
Positive emotions (0-12)	1954	12.2

Note. n=16064. Baseline year=2002.

Supplementary Table 2. Comparison of Baseline Variables between Analytical Sample and Drop-out Sample.

**Variables.**	Univariable logistic		Multivariable logistic	
	OR	p value	OR	p value
Social engagement	1.48	<.001	1.11	<.001
**Sociodemographic characteristics**				
Age				
65-74	Ref.	-	Ref.	-
75-84	0.56	<.001	0.68	<.001
85+	0.16	<.001	0.30	<.001
Sex				
Woman	Ref.	-	Ref.	-
Man	1.24	<.001	0.85	<.001
Ethnicity				
Minority	Ref.	-	Ref.	-
Han	0.74	<.001	0.89	0.193
Occupational status				
Low level	Ref.	-	Ref.	-
High level	1.38	<.001	1.06	0.458
Education years				
0	Ref.	-	Ref.	-
1-5	1.38	<.001	0.97	0.537
6-9	1.64	<.001	1.01	0.913
10+	1.35	<.001	0.89	0.283
Residence				
Rural	Ref.	-	Ref.	-
Urban	0.84	<.001	0.85	<.001
Region				
Eastern China	Ref.	-	Ref.	-
Central China	0.80	<.001	0.88	0.029
Western China	0.67	<.001	0.69	<.001
Sufficient financial support				
No	Ref.	-	Ref.	-
Yes	1.01	0.866	0.96	0.436
Pension				
No	Ref.	-	Ref.	-
Yes	1.27	<.001	0.89	0.047
**Health status**				
ADL (0-6)	0.60	<.001	0.75	<.001
Chronic diseases numbers (0-11)	0.97	0.225	0.93	0.012
MMSE score (0-30)	1.09	<.001	1.04	<.001
Positive emotions (0-12)	1.10	<.001	1.03	<.001

Note. OR= odds ratio; Ref.=reference. The numbers of analytical sample and drop-out sample were 8117 and 7947, respectively. Univariable and multivariable logistic models were used, and the drop-out sample was set as the reference. Baseline year=2002.

Supplementary Table 3. Marital Status of Older Adults between Three Age Groups.

Variables.	Total (n=8117)	Unmarried (n=4913)	Married (n=3204)	p value
	N (%)	N (%)	N (%)	
Age				
65-74	2530 (31.2)	822 (16.7)	1708 (53.3)	<.001
75-84	2401 (29.6)	1398 (28.5)	1003 (31.3)	
85+	3186 (39.3)	2693 (54.8)	493 (15.4)	

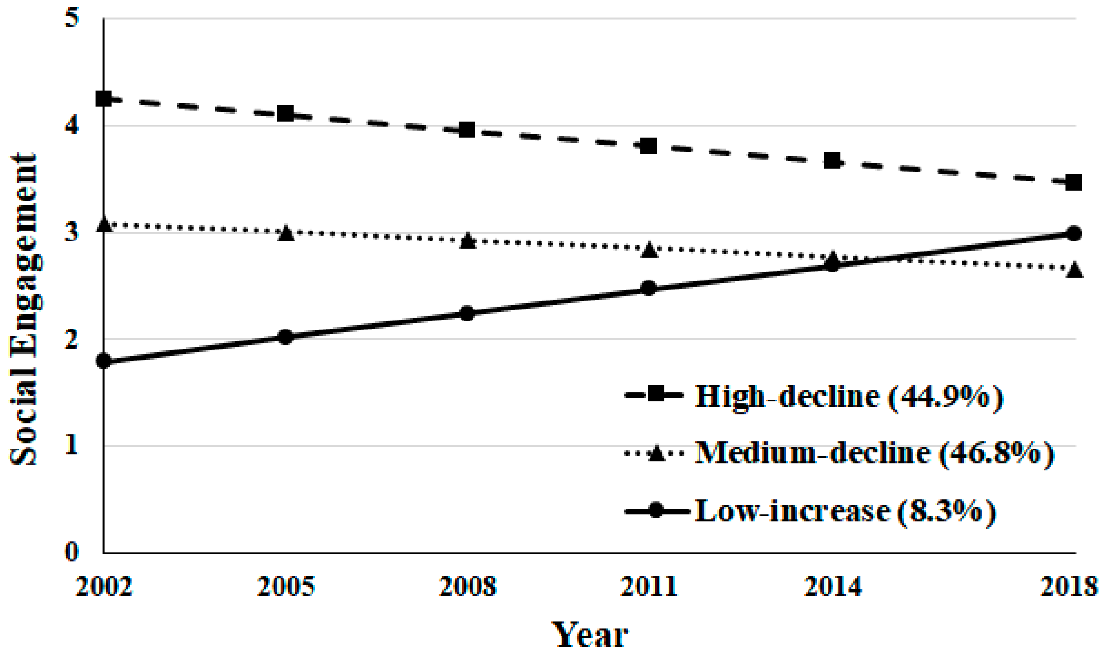

Figure S1. Heterogenous trajectory classes of social engagement for older adults with complete information for at least three waves (n= 4159). The three class GMM was selected as final model (sample-size adjusted Bayesian information criteria= 39327.6; entropy= 0.75; Vuong-Lo-Mendell-Rubin Likelihood Ratio Test p value< .001), including “high-decline” class (intercept= 4.25, p< .001; slope= -0.15, p<.001), “Mow-decline” class (intercept= 3.09, p<.001; slope= -0.08, p<.001), and “low-increase” class (intercept= 1.79, p< .001; slope= 0.23, p<.001). It showed similar pattern of social engagement trajectory classes with our analytic sample (n= 8117).

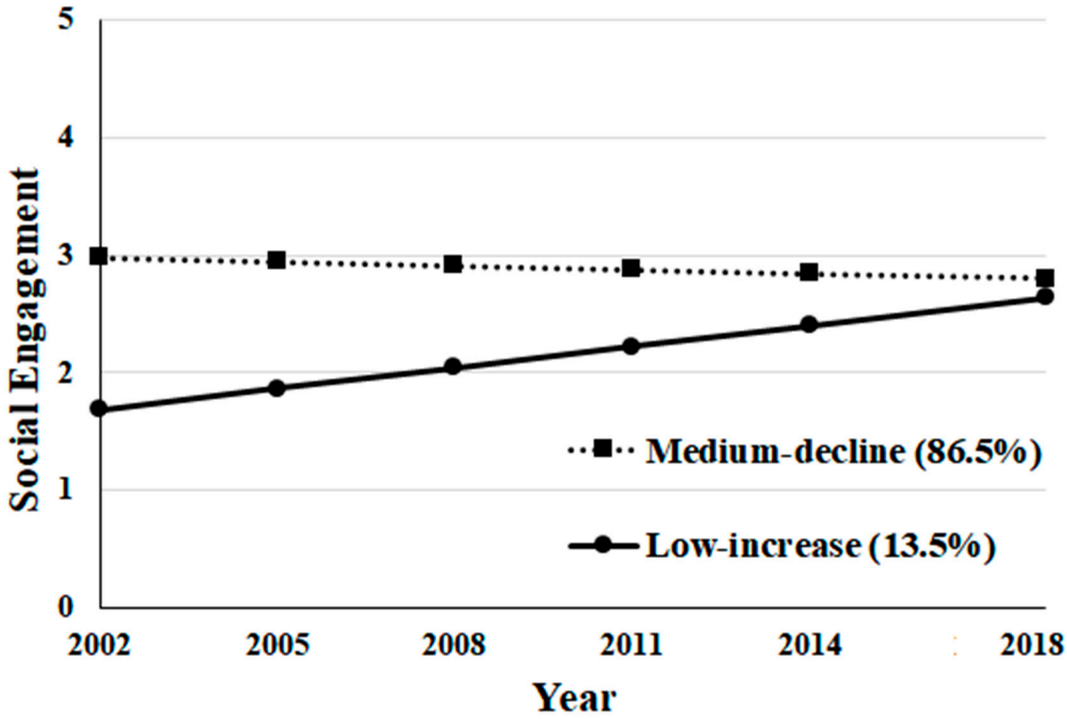

Figure S2. Heterogenous trajectory classes of social engagement for older adults with different definition of social engagement by excluding spouse from cohabitant or confidant (n= 5160). The two class GMM was selected as final model (sample-size adjusted Bayesian information criteria= 26033.8; entropy= 0.80; Vuong-Lo-Mendell-Rubin Likelihood Ratio Test p value<.001), including "Medium-decline" class (intercept= 2.98, p<.001; slope= -0.03, p<.001) and "low-increase" class (intercept= 1.68, p<.001; slope= 0.18, p<.01). The "high-decline" class of social engagement trajectory in original sample was not identified here. Compared to exclude spouse from cohabitant or confidant in this definition of social engagement, married people who were more dependent on their spouse may had higher level of social engagement than others in our original definition. However, two classes of change patterns (declining and increasing) of social engagement still existed.
